# Proteomic Identification of Dengue Virus Binding Proteins in *Aedes aegypti* Mosquitoes and *Aedes albopictus* Cells

**DOI:** 10.1155/2013/875958

**Published:** 2013-11-10

**Authors:** Maria de Lourdes Muñoz, Gustavo Limón-Camacho, Rosalinda Tovar, Alvaro Diaz-Badillo, Guillermo Mendoza-Hernández, William C. Black

**Affiliations:** ^1^Department of Genetics and Molecular Biology, Centro de Investigación y de Estudios Avanzados del Instituto Politécnico Nacional, 07360 Mexico, DF, Mexico; ^2^Coordinación Academica, Universidad Autónoma de la Ciudad de México, 06720 Mexico, DF, Mexico; ^3^Department of Biochemestry, Faculty of Medicine, Universidad Nacional Autonoma de México, Edificio de Investigación, 04510 Mexico, DF, Mexico; ^4^Department of Microbiology, Immunology and Pathology, Colorado State University, Fort Collins, CO 80523-0015, USA

## Abstract

The main vector of dengue in America is the mosquito *Aedes aegypti*, which is infected by dengue virus (DENV) through receptors of midgut epithelial cells. The envelope protein (E) of dengue virus binds to receptors present on the host cells through its domain III that has been primarily recognized to bind cell receptors. In order to identify potential receptors, proteins from mosquito midgut tissue and C6/36 cells were purified by affinity using columns with the recombinant E protein domain III (rE-DIII) or DENV particles bound covalently to Sepharose 4B to compare and evaluate their performance to bind proteins including putative receptors from female mosquitoes of *Ae. aegypti*. To determine their identity mass spectrometric analysis of purified proteins separated by polyacrylamide gel electrophoresis was performed. Our results indicate that both viral particles and rE-DIII bound proteins with the same apparent molecular weights of 57 and 67 kDa. In addition, viral particles bound high molecular weight proteins. Purified proteins identified were enolase, beta-adrenergic receptor kinase (beta-ARK), translation elongation factor EF-1 alpha/Tu, and cadherin.

## 1. Introduction

Dengue fever, dengue hemorrhagic fever (DHF), and dengue shock syndrome (DSS) are the most important arthropod-borne diseases nowadays, affecting people living mainly in tropical and subtropical regions, where environmental conditions favor the proliferation of the mosquito vector *Ae. aegypti*, as this has been spread to other regions in the world likely due to gradual climatic changes [1, 2]. Though, this may contribute to the spread of this disease, this has not been demonstrated [3].

The etiological agent of dengue is a positive-stranded RNA virus containing 3 structural proteins (C, prM, E) and 7 nonstructural proteins (NS1, NS2A, NS2B, NS3, NS4A, NS4B, and NS5). It belongs to the family *Flaviviridae*, genus *flavivirus*, known as dengue virus (DENV), and includes serotypes from 1 to 4. Each serotype is also classified into a series of genotypes or subtypes [3–6]. Dengue virus genotypes differ in virulence, including their human pathogenicity and epidemic potential.

Dengue virus is transmitted to humans in America mainly by the mosquito vectors *Aedes aegypti* [7] infecting primary human cells such as peripheral blood leukocytes, blood monocytes/macrophages, dendritic cells, and B lymphocytes [7]. Dengue virus attaches to the host epithelial cell receptors protein E-mediated [8, 9] and enters the cell mainly via this receptor by clathrin-dependent endocytosis [10–13].

In mammalian cells, several DENV receptors have been described [14–19] as well as in mosquito cells; however the molecular identity of the receptors in mosquito cells has not been completely elucidated. The apparent molecular weights described for these proteins are between 20 to 40 kDa and 57 to 130 kDa in size and bind dengue virus particles *in vitro* [8, 20–24]. In addition, Mercado-Curiel et al. [23] reported that specific antibodies against the membrane proteins R67 and R80 inhibited infection of C6/36 cells. Further, a protein with molecular mass of 57 kDa was also purified by affinity chromatography using a DEN2-Sepharose 4B column [23].

Viral envelope (E) protein of DENV as other *Flavivirus* has a homology of about 40% among different members of the family [25], and the crystal structures of this protein revealed three domains (I, II, and III) containing significant structural conservation [26–28]. DENV E protein is a class II fusion protein responsible for host cell attachment, entry, and virus-mediated cell membrane fusion.

It has also been shown that domain III of the envelope glycoprotein is an immunoglobulin-like structure and that the main viral region interacts with receptors on the host cells [29–36]. It has been also demonstrated that EIII domain of DENV-2 inhibits infection of DV on C6/36 cells and mammalian cells, suggesting that EIII domain binds molecules on the cell membrane that may participate in receptor-mediated DV entry [37]. However, the molecular mechanism of DENV-receptors has not been characterized in mosquitoes until now. We would expect that such molecular interactions in mosquito vector would influence virus passage through the different mosquito barriers: the first one is that after the virus established a midgut (MG) infection (MI) by overcoming the MG infection barrier (MIB): next replication in the MG epithelium, and then that virus must pass through a MG escape barrier (MEB) and replicate in other tissues to establish a disseminated infection (DI). Finally, virus must infect salivary glands and be shed in the saliva to be transmitted to a vertebrate host [38, 39]. 

It is accepted that the mechanisms by which DENV infects its target host cell should be the major determinant of the virus cellular tropism and critical for viral pathogenesis. Erb et al. [37] demonstrated that the FG loop located in DIII, where DENV2 has an extended loop motif between the F and G beta strands, was critical for the infection of *Aedes aegypti* mosquito MGs and mammalian cells by mutational studies. In addition, Butrapet et al. [40] identified critical amino acids within the hinge region of DENV-2, that are vital for virus fusion and replication. While domain III has already been used to study immunological and pathological mechanisms [40–46], its suitability for isolating specific receptors has not yet been investigated; then, we are showing in this communication the purification of DENV binding proteins from *Ae. aegypti* MG tissue and C6/36 cells by affinity chromatography using particles of DENV-2, -1, and -4 or rE2-DIII covalently bound to Sepharose 4B. In addition the identity of these proteins was determined by proteomic analysis.

## 2. Materials and Methods

### 2.1. Virus

DENV-2 Jamaica was expanded in Vero cells [47], purified from the culture supernatants as previously described [48], and kept frozen at −70°C until use. Briefly, Vero cells were cultured at 37°C, 2% CO_2_ in Dulbecco's Modified Eagle's Medium (DMEM; HyClone, Logan, Utah, USA) supplemented with 5% heat-inactivated fetal bovine serum (FBS; Gibco BRL, Gaithersburg, MD, USA), 100 units/mL of penicillin, and 100 *μ*g/mL of streptomycin. Vero cells (2 × 10^6^/100 mm plate) were infected with 0.2 mL of DENV-2 inoculums with an input MOI of 600 PFU/plate and incubated for 10 days. We also included in our studies DENV-1 Hawaii, DENV-2 S1656OAX05 (Asian/American genotype), DENV-3 H-87, and DENV-4 H-341 strains.

### 2.2. Virus Purification

Viruses were purified on sucrose gradients essentially as described by Srivastava et al. [49] with minor modifications previously described [23]. The virus was recovered, suspended in PBS, and kept frozen at −70°C until use. The titer of the viral stock was adjusted to 6 × 10^6^ PFU/mL. Virus purity was examined for total protein and by RT-PCR and transmission electron microscopy. 

### 2.3. Virus Biotinylation

NHS-coupled biotinyl compounds have been used to label cell surface proteins previously [8]. The procedure to biotinylate DENV particles was as follow. Briefly, dissolve (+)-Biotin N-hydroxysuccinimide ester (Sigma, Catalog Number H1759) in DMSO immediately prior to use protecting solution from the light at a concentration of 11 mg/mL. Purified viruses were biotinylated by suspension of the viral pellet (2 mg of protein) in 0.8 mL of ice cold Phosphate buffer, pH 7.5 (PB) by adding 0.2 mL of NH-D-Biotin solution with gentle stirring and incubated overnight at 4°C or 3 h at ambient temperature. Biotinylated virus recovered after centrifugation at 100,000 ×g for 2 h was suspended in PBS (500 *μ*L). The degree of biotinylation was determined by dot blot, and the viral stock was kept at −70°C until use [8]. 

### 2.4. Mosquito Culture

*Aedes aegypti* mosquitoes from the strains DS3 (susceptible to DENV), IBO-11 (refractory to infection), DMEB (midgut escape barrier), and Mori (collected in Monterrey, México) were laboratory-reared and maintained at 32°C and 80% RH with a 12 h photoperiod using standard mosquito-rearing procedures [50]. The entire MG was dissected from more than 1500 mosquitoes at day 5 after egg hatching. The procedure was carried out in 10 *μ*L phosphate buffered saline (PBS). After dissection, each MG was rinsed twice in the same solution, quickly removed, and snap-frozen at −70°C until use.

### 2.5. Protein Extract Preparation

To optimize MG protein extraction, frozen MGs were homogenized in buffer E (0.05 M Tris-HCl, pH 7.2, 1 mM EDTA), containing 1 *μ*L/mL of protease inhibitor cocktail (Sigma P9599) and 0.01, 0.05, 0.1, 0.5, or 1.00% v/v of Triton X-100. Protein extracts were centrifuged for 10 min at 29000 ×g at 4°C. Total protein concentration was determined as described previously by Bradford [51].

### 2.6. Virus Overlay Protein Binding Assay (VOPBA)

Mosquito MG proteins were separated by 10% SDS-PAGE according to the method described by Laemmli [52] and blotted onto PVDF membranes (BioRad) by Towbin's technique [53]. The procedure was followed as previously described [8]. Previous results in our laboratory have showed that biotinylated virus recognized the same proteins as compared to virus without any treatment [8].

### 2.7. Affinity Chromatography

To perform the affinity chromatography assays, DENV-2, -1, -4 (5.7 × 10^8^ PFU/mL), or domain III of E protein (500 *μ*g, rE2-DIII) (ProSpec-Tany TechnoGene LTD) were covalently bound to 1 mL of CNBr-activated Sepharose 4B as recommended by the manufacturer (Amersham Biosciences) as described elsewhere [23]. Both affinity columns were stored in 0.002% sodium azide at 4°C until use.

Midgut protein extract obtained as described above (300 *μ*g) was applied to the DENV-Sepharose 4B column, or rE2-DIII-Sepharose 4B column equilibrated in Buffer E containing 0.5 M NaCl, and washed with the same buffer thoroughly. The DENV-2 binding proteins were eluted with 0.1 M glycine-HCl pH 2.7 or buffer E containing 1 M NaCl. Fractions of 0.500 mL were collected, and the protein concentration was monitored by the Bradford method [51]. Eluted proteins in each fraction were concentrated by acetone-precipitation [54], separated by 10% SDS-PAGE (sodium dodecyl sulfate polyacrylamide gel electrophoresis) [52] and Coomassie Brilliant Blue or silver stained [55]. Total protein extracts from *Ae. aegypti* mosquito DS3 (susceptible to DENV), IBO-11 (refractory to infection), and DMEB (the membrane escape barrier infected exclusively in the midgut epithelial cells) strains were also separated by SDS-PAGE, and then the proteins that migrated as the purified proteins (57 and 67 kDa) were also excised from the gels and the proteomic analysis was carried out (Table 1). Protein assignment was done by at least two peptide matches.

### 2.8. Protein Sequencing

The protein bands of interest were excised from a Coomassie Brilliant Blue R-250-stained gel, digested with trypsin, and identified by mass spectrometry (3200 TRAP hybrid tandem mass spectrometer, Applied Biosystem/MDS Sciex, Concord, ON, Canda). LC/MS/MS analysis of tryptic peptides was carried out using a NanoAcquity ultraperformance liquid chromatograph (UPLC) (Waters Corporation), coupled to a Q-ToF Synapt High Definition Mass Spectrometer (Waters Corporation), and equipped with a NanoLockSpray ion source. Protein identification was performed from the MS/MS spectra data sets using the MASCOT search algorithm (Version 1.6b9, Matrix Science, London, UK) available at http://www.matrixscience.com/ [56]. Peptide mass tolerance was set to ±1.2 Da and fragment mass tolerance to ±0.6 Da and the taxonomy parameter set to all species. Each MS/MS spectrum was also searched for *Ae. aegypti* against the data sets at VectorBase [57].

## 3. Results

**Figure 1:**
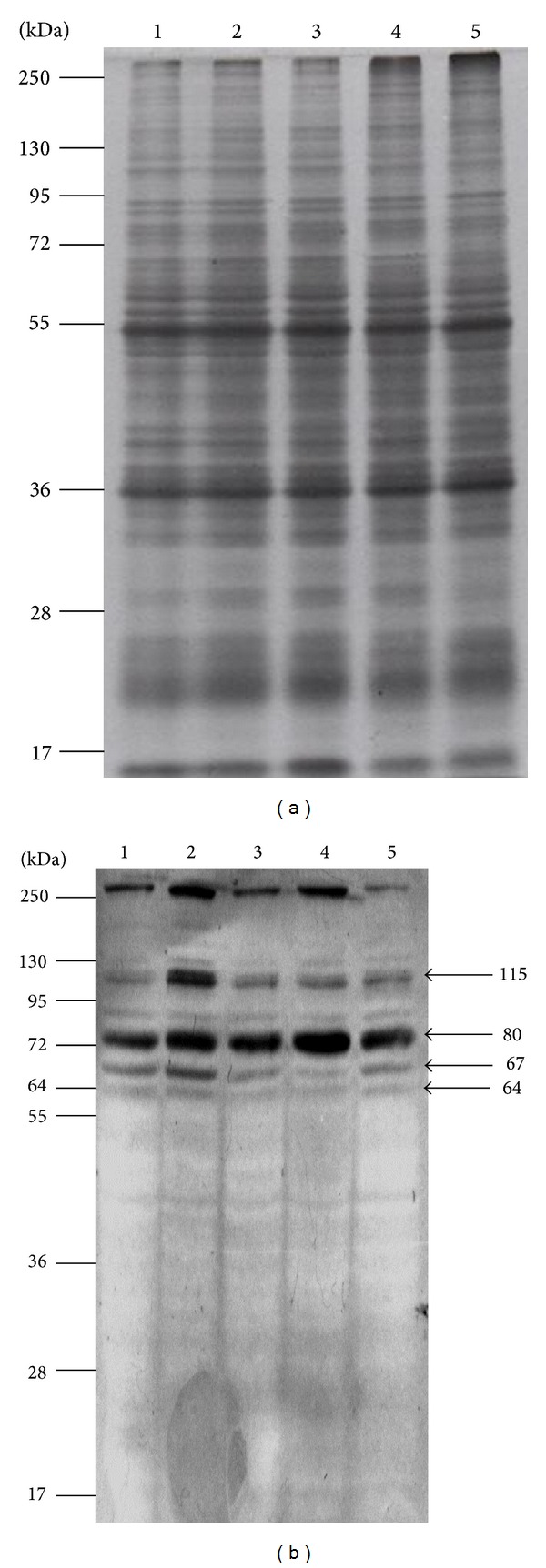
Midgut total protein extraction with Triton X-100 and VOPBA. (a) Proteins were extracted from mosquito MG tissue at different Triton X-100 concentrations, separated by SDS-PAGE, and stained with Coomasie Blue. Triton X-100 concentrations were 0.01, 0.05, 0.1, 0.5, and 1% corresponding to lane 1 to 5, respectively. (b) Proteins, separated by SDS-PAGE, were blotted onto PVDF and incubated with biotinylated DENV-2 and then with AP-Streptavidin. Proteins recognized by DENV-2 were developed with BCIP/NBT according to the procedure previously described [8]. The apparent molecular weights of these proteins are shown on the right side of (b). Molecular weight markers are shown on the left side in (a) and (b).

To optimize solubilization of membrane proteins from mosquito MGs tissue was homogenized with buffer E containing Triton X-100, 0.01, 0.05, 0.1, and 0.5 or 1.00% v/v. Each protein extract was separated by SDS-PAGE and stained with Coomasie Blue.  Figure 1(a) shows protein integrity and the same protein pattern at all Triton X-100 concentrations. To detect DENV-2 binding proteins, MG protein extracts were separated by SDS-PAGE, blotted onto a PVDF membrane, and incubated with biotinylated DENV-2 as mentioned in the Materials and Methods section.  Figure 1(b) displays the proteins recognized by DENV-2 labeled with biotin. The optimal concentration of Triton X-100 to extract maximal DENV binding protein amount was 0.05% v/v (Figure 1(b), lane 2), since protein bands revealed by DENV-2 labeled with biotin are of greater intensity. Four major proteins with molecular masses of 57, 67, 80, and 115 kDa were observed in all lines (Figure 1(b)). Extraction of proteins with apparent molecular weight of 67 and 115 (Figure 1(b), lane 2) with the buffer containing 0.05% v/v Triton X-100 displayed higher densities, suggesting higher concentrations. This suggests that both proteins may be located at the membrane. Consequently, protein extraction was subsequently performed at a concentration of 0.05% Triton X-100. Negative control without virus showed no bands (data not shown).

**Figure 2:**
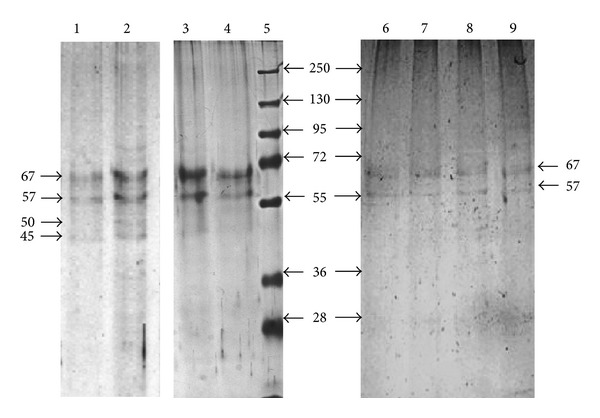
Affinity chromatography of C6/36 cell extracts. Proteins were purified from C6/36 cells by affinity chromatography using DEN-2, -1, -4, or rE2-DIII-Sepharose 4B column as described in the methods section. Aliquots of 500 *μ*L were collected from each column and proteins were acetone-precipitated. Proteins eluted from DENV-2-Sepharose 4B columns with buffer E containing 1 M NaCl are displayed in lines 1 and 2, or 0.1 M Glycine pH 2.7 in lines 3 and 4. Proteins eluted from rE2-DIII-Sepharose 4B column with 0.1 M Glycine pH 2.7 are displayed in lines 6–9. Proteins were separated by 10% SDS-PAGE and Coomassie Brilliant Blue or silver stained. The apparent molecular weights of these proteins are shown on the right side. Molecular weight markers (line 5) are shown on the left side.

In order to recover all proteins bound to the affinity column, after passing protein extracts from C6/36 cells through DENV-2-Sepharose 4B column, the proteins were eluted from independent columns with buffer E containing 1 M NaCl (Figure 2, lines 1 and 2), or 0.1 M Glycine pH 2.7 (Figure 2, lines 3 and 4).

Once the protein extraction procedure was optimized, dengue virus binding proteins were purified by affinity chromatography by passing protein extracts from C6-36 cells through a rE2-DIII-Sepharose 4B column and eluted with 0.1 M Glycine pH 2.7 containing 0.5 M NaCl (Figure 2, lines 6–9). Proteins with apparent molecular weights of 57 and 67 were mainly eluted with this column (Figure 2). 

**Figure 3:**
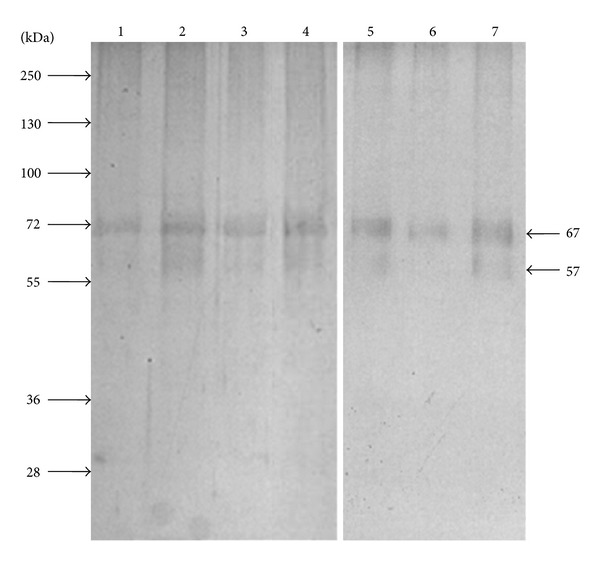
Affinity chromatography of MG protein extracts. MG proteins were purified from extracts of different *Ae. aegypti* strains (DMEB, DS3, IBO-11, or Mori) by affinity chromatography using DENV-2, -1, -4 or rE2-DIII-Sepharose 4B column as described in Section 2. Midgut proteins were eluted from DENV-2-Sepharose 4B columns with buffer E containing 1 M NaCl (lines 1-2), or 0.1 M Glycine pH 2.7 (lines 3-4) and from rE2-DIII-Sepharose 4B column with 1 M NaCl (line 5) or 0.1 M Glycine pH 2.7 containing 0.5 M NaCl (line 6-7). Aliquots of 500 *μ*L were collected from each column and proteins were acetone-precipitated and separated by 10% SDS-PAGE and Coomassie Brilliant Blue or silver stained. The apparent molecular weights of these proteins are shown on the right side. Molecular weight markers are shown on the left side.

Then, dengue virus binding proteins were purified by affinity chromatography by passing protein extracts from *Ae. aegypti* MG through a DENV-2 or rE2-DIII-Sepharose 4B columns (Figure 3). Representative patterns of MG proteins retained and eluted from the column (from at least four experiments) are shown in Figure 3. Proteins with apparent molecular weights of 57, 67 kDa were eluted with buffer E containing 1 M NaCl (Figure 3, lines 1-2) or 0.1 M Glycine pH 2.7 (Figure 3 lines 3-4) from DENV-2 Sepharose 4B column. Proteins showing the same apparent molecular weights were eluted from rE2-DIII-Sepharose 4B column with buffer E containing 1 M NaCl (Figure 3, lines 5) or 0.1 M Glycine pH 2.7 (Figure 3, lines 6-7). The eluted proteins (EP) were stored at −70°C for a further analysis.

### 3.1. Identification of Mosquito Proteins That Interact with Dengue Virus

Proteins identified from the MS/MS spectra data sets using the MASCOT search algorithm [56] with trypsin enzyme specificity are shown in Table 1. Peptide sequence of each protein is displayed in Table 2. Proteins are ordered from the top to the bottom for the number of peptides identified as well as for the number of the experiments. Proteomic analysis was performed in protein extract purified by the affinity columns or separated by SDS-PAGE and then excised from the gel.

**Figure 4:**
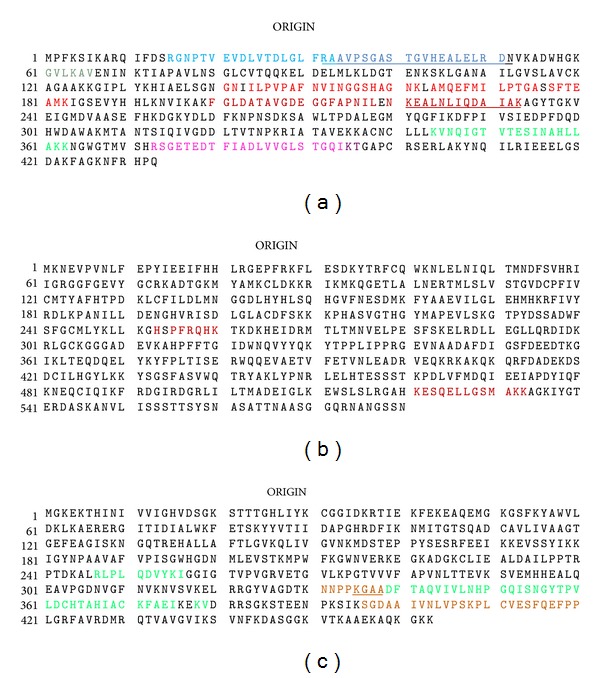
Identification of enolase, beta-adrenergic receptor kinase, and translation elongation factor EF-1 alpha/Tu as DENV-binding proteins by LC MS/MS analysis of the excised protein bands corresponding to the apparent molecular weights of 57 and 67 kDa. The colored sequences represent the amino acid peptides identified as enolase (a), beta-ARK (b), and translation elongation factor EF-1 alpha/Tu (c) using MS/MS spectrometry after in-gel digestion of the protein-staining band (Tables 1 and 2). The protein sequence refers to gi*|*157121051*|*ref*|*XP_001653750*|*, gi*|*157114479*|*ref*|*XP_001652291*|*, and gi*|*94468780*|*gb*|*ABF18239.1*|*, respectively.

**Figure 5:**
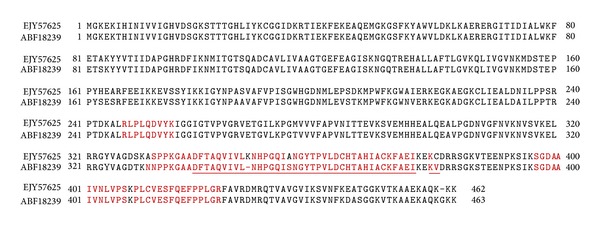
Amino acid sequence analysis of two different translation elongation factors EF-1 alpha/Tu. Alignment of these elongation factors (EJY57625 and ABF18239) identified in C6/36 cells, and MGs of *Ae. aegypti* mosquitoes of the IBO-11 and MORI strains is shown. Identified peptides are shown in red color.

Proteomic analysis of proteins obtained from total protein extracts of *Ae. aegypti* mosquito DS3, IBO-11, and DMEB strains separated by SDS-PAGE that migrated as the purified proteins (57 and 67 kDa) with at least two peptide matches is shown in Table 1. The proteins identified were enolase, beta-ARK, translation elongation factor EF-1 alpha/Tu, and cadherin. Translation elongation factor EF-1 alpha/Tu and cadherin had been identified previously, thus ensuring that the procedure described in this work is suitable to the identified proteins bound to DENV and E protein domain III. Peptide sequence AKPGAEAHPPFRQHK has partial alignment with beta-ARK (ref*|*XP_001652291*|*) and with ATP-dependent RNA helicase (ref*|*XP_001648042.1*|*); however, the identification of beta-ARK was confirmed by the match of ESQELLGSMAKK peptide with beta-ARK identified in two mosquito strains (DS3 and DMEB). Although, cadherin is showing only one peptide, the peptide match to this protein has a very high score of 52 (16/17 amino acids). Proteins identified in C6/36 cells or mosquito MGs from DMEB, DS3, IBO-11, or Mori strains are also included in Table 1. Manual analysis was used to confirm peptide identity (Figure 4). Peptide sequence coverage was 35% for enolase, 2.6% for beta-ARK, and 20% for translation elongation factor EF-1 alpha/Tu. Because translation elongation factor EF-1 alpha/Tu matched two proteins, we manually verified mass spectra for presence of unique peptides for each homologous assignment. In Figure 5 we demonstrate the alignment for these two homologous proteins EJY57625 and ABF18239 and peptides identified in each of those two proteins. Peptides NNPPKQAA and K.GASDFTAQVIVLNHPGQIANGYTPVLDCHTAVIACK-FAEIQQK.V were specific for protein EJY57625 (Figure 5).

## 4. Discussion

*Flavivirus* vector competence studies in *Ae. aegypti* have indicated that the MIB is a major determinant of transmission [58, 59] and have shown wide variation among *Ae. aegypti* populations and *flaviviruses* including DENV [9, 38, 60]. Studies on mosquito receptors have displayed protein receptors on MG epithelial cells that may be the base to develop a strategy to control mosquito vector through blocking virus infection. In order to elucidate the nature of these receptors, mass spectrometry-based proteomic analysis of the purified proteins was performed. In our study, we are showing the isolation of proteins by affinity columns bound to the virus or domain III of the E protein of dengue 2 virus. Considering that *Ae. aegypti* MG is the best candidate to disrupt the virus life cycle within the mosquito because it is the earliest interface between insect and virus and that DENV attachment to MG epithelial cell receptors is also critical for understanding the initial virus-vector interactions, this will help to explain MIB to DENV infection and variations in vector competence.

Accordingly, identification of viral receptors in the MG would represent a critical step in understanding vector competence and designing possible targets for preventing viral entry to cells and therefore inhibiting the infection. Published data have shown that domain III of the viral E protein is involved in target cell recognition [29] and binding of host cell surface receptors [32, 34–37]. Consequently, identification of dengue virus binding proteins by affinity chromatography using rE2-DIII will help to understand virus cell entry and to design strategies to block virus infection in the mosquito cells. Thus, in order to purify DENV binding proteins, rE2-DIII or viral particles were covalently bound to Sepharose 4B matrix.

Our results suggest that purified proteins by rE2-DIII-Sepharose 4B affinity column correspond to the same proteins purified by dengue particles with apparent molecular weights of 57 and 67 that were also consistently and previously reported in C6/36 cell membranes [23]. Specific antibodies against the 67 kDa protein inhibited virus infection [8, 23]. Although, DENV-Sepharose 4B bound additional proteins, we focused our studies to the proteins with apparent molecular weight of 57 and 67 bound to DENV particles and E protein domain III (Figures 2 and 3, Table 1). We also showed that DENV-1, -2, and -4 bound the same proteins with apparent molecular weights of 57 and 67 kDa.

These results are very important since the identity of specific MG mosquito proteins bound to viral particles and domain III of E protein has not been previously reported. The proteins identified by the proteomic analysis were enolase, elongation factor 1, beta-ARK, and cadherin. Enolase is a glycolytic enzyme and has been found in small vesicles outside the cell [61, 62]; it binds to plasminogen and helps pathogens to invade [63]. Enolase is also found in viral particles [64–66] and is required for the transcription of Sendai virus [67]. Furthermore, enolase has been identified in the MG brush border of *Ae. aegypti* mosquitos [68]. We identified enolase in protein extracts of C6/36 cell, and in the MG of *Ae. aegypti* mosquitoes from DS3 and DMEB strains and also showed that this protein is bound to DENV-2. In our previous reports we established that the 67 kDa protein is a membrane DENV binding protein [8, 9]. Therefore, our results agree with previous reports as enolase is in the brush border of mosquito MGs [68]; This reinforces the idea that enolase may be a DENV receptor of *Ae. aegypti* MGs. In addition, enolase has been also reported to bind to West Nile and DENV virus envelope and capsid proteins, respectively [69].

The second protein identified by the proteomic analysis was the beta-ARK with apparent molecular weight of 67,000. This protein specifically phosphorylates the agonist-occupied form of the beta-adrenergic and closely related receptors, probably inducing a desensitisation of them in higher eukaryotic organisms. This kinase is a member of the G protein-coupled receptor kinase (GRKs) family and catalyzes the phosphorylation of the activated forms of the beta-adrenergic receptor (beta-AR). As member of GRKs, this protein is also very important, because it has been implicated in the specific phosphorylation on membrane protein receptors and in the regulation of signal transduction mechanisms [70]. Furthermore, beta-ARK also may help virus endocytosis facilitating receptor endocytosis, similarly to beta-ARK reported to directly interact with phosphoinositie-3-kinase (PI3K) promoting its membrane localization, phosphoinositide production, AP-2 adaptor protein recruitment to the receptor, and receptor endocytosis [71]. This protein was identified in C6/36 cells and DMEB, and DS3 *Ae. aegypti* mosquito strains. 

The translation elongation factor EF-1 alpha/Tu was the third identified protein in C6/36 cells and MGs of *Ae. aegypti* mosquitoes of the DMEB and IBO-11 strains purified by affinity chromatography using DENV-2 and -4. Previously, this protein was also identified as an NS4 binding protein of DENV and WNV [69]. Furthermore, it has been also reported that DENV envelope protein binds to cadherin [69]. Furthermore, cadherin identified in this work has also been reported to bind to DENV envelope protein [60].

The data in the present paper strongly support that enolase may be a receptor for DENV-2, in MG cells from *Ae. aegypti,* and this protein may correspond to the 57 or 67 kDa protein previously reported [8, 9]. Differences in molecular weight mass may be due to posttranslational modifications, residual protease activity, or association with other molecules as has been formerly reported.

In addition, the procedure described here may be very useful in future studies to determine the proteins that bind to different domains of E protein or to other viral proteins. To the best of our knowledge, this is the first paper that displays a method to purify *Ae. aegypti* MG proteins by affinity chromatography by means of viral particles compared to rE2-DIII and establish the identity of the proteins with apparent molecular weights of 57 and 67 kDa. 

## 5. Conclusions

This study identified enolase, beta-ARK, translation elongation factor EF-1 alpha/Tu, and cadherin mosquito as binding proteins that may play important roles as host factors during viral infection of mosquito cells. Enolase, beta-ARK, and cadherin may serve as DENV receptors, and translation elongation factor EF-1 alpha/Tu may be very important during virus replication. All proteins were identified in C6/36 cells and in the *Ae. aegypti* DS3, DMEB, and IBO-11, and Mori strains that differ in their vector competence for DENV; then we are suggesting that all mosquito strains of *Ae. aegypti* and C6/36 cells from *Ae. albopictus* interact probably with the same protein domain. In addition, the protein with the same apparent molecular weight was bound by DENV-1, -2, and -4 and rE2-DIII. Future studies will be necessary to determine the specific role of each protein in each strain to know how they participate in vector competence. 

## Figures and Tables

**Table 1:** Mosquito proteins bound to DENV.

DENV-Sepharose 4B*	Cell/tissue expression/gel slice**	Name****	Accession number	Accession swissprot	Predicted mass (Da)	Size (aa)	Mascot score	Theoretical isoelectric point*	Number of peptides	Protein coverage (%)
DENV-2	C6/36 57 kDa	Enolase^1^	gi∣157121051∣ref∣XP_001653750∣	Q17KK5	46621	433	76.1	5.6	4	15.9
DENV-2	C6/36 67 kDa	Enolase^2^	gi∣157121051∣ref∣XP_001653750∣	Q17KK5	46621	433	60.5	5.6	1	7.4
DENV-2	DS3 (extract)	Enolase^3^	gi∣157121051∣ref∣XP_001653750∣	Q17KK5	46621	433	20.2	5.6	1	1.4
None	DMEB*** 67 kDa	Enolase^4^	gi∣157121051∣ref∣XP_001653750∣	Q17KK5	46621	433	75	5.6	2	13
DENV-1	DMEB and DS3 67 kDa	Beta-adrenergic receptor kinase^1^	gi∣157114479∣ref∣XP_001652291∣	Q174J9	66217	580	40/26.9	6.74	1	2.6
DENV-4	C6/36 67 kDa	Beta-adrenergic receptor kinase^2^	gi∣157114479∣ref∣XP_001652291∣	Q174J9	66217	580	40/26.9	6.74	1	2.6
DENV-2	C6/36 57 kDa	Translation elongation factor EF-1 alpha/Tu^1^	gi∣94468780∣gb∣ABF18239.1∣	Q1HR88	50,473	463	82.9	9.61	1	5.4
DENV-2	MORI (extract)	Translation elongation factor EF-1 alpha/Tu^2^	gi∣94468780∣gb∣ABF18239.1∣	Q1HR88	50,473	463	21.8	9.61	1	1.7
None	IBO-11 57 kDa	Translation elongation factor EF-1 alpha/Tu^3^	gi∣94468780∣gb∣ABF18239.1∣	Q1HR88	50,473	463	63	9.61	2	10.8
DENV-4	C6/36 80 kDa	Cadherin	gi∣157115805∣ref∣XP_001658290∣	Q17LY6	186427	1653	52.0	4.74	1	1%

*Affinity chromatography was performed with DENV-2, -1, or -4.

**Bands of interest were excised at the molecular weight of interest (57 or 67 kDa).

***Total extract of MGs from *Ae. aegypti* mosquitoes strain DMEB was separated by SDS-PAGE and the band with apparent molecular weight of 67 kDa was excised for a further analysis.

****Superscript number in the name of the protein indicates the number of the experiment.

**Table 2:** Distinct host peptides identified by mass spectrometry bound to DENV.

Cell/tissue expression	Protein name	Experment number	Peptide identified	Score
C6/36 (57 kDa)	Enolase	1	K.EALNLIQDAIAK.A	45.6
			R.GNPTVEVDLVTDLGLFR.A	62.1
			K.VNQIGTVTESINAHLLAK.K	76.1
			R.SGETEDTFIADLVVGLSTGQIK.T	76.1
C6/36 (67 kDa)		2	FGLDATAVGDEGGFAPNILNNKEALDLINEAISK	60.5
DS3		3	GVLKAVTQ	20.2
DMEB (67 kDa)		4	R.AAVPSGASTGVHEALELR.D	53.2
			K.NLILPVPAFNVINGGSHAGNKQAMQEFMILPTGACSFTEAMK.M	21.7

DMEB (67 kDa)	Beta-adrenergic receptor kinase	1	ESQELLGSMAKK	40.1
DS3 (67 kDa)		2	ESQELLGSMAKK	40.1
C6/36 (67 kDa)		3	AKPGAEAHPPFRQHK	26.9

C6/36 (57 kDa)	Translation elongation factor EF-1 alpha/Tu	1	SGDAAIVNLVPSWPLCVESFQEFPPLGR	82.9
Mori (extract)		2	NNPPKQAA	21.8
IBO		3	K.GASDFTAQVIVLNHPGQIANGYTPVLDCHTAVIACKFAEIQQK.V R.LPLQDVYK.I	63

C6/36 (80 kDa)	Cadherin	1	FLIDYGSGTLELRIATK	52

*Proteomic analysis was performed in protein from C6/36 cells, mosquito MGS purified by affinity chromatography (extract), or in the bands of interest excised after separation by SDS-PAGE.
